# New Insights into Handling Missing Values in Environmental Epidemiological Studies

**DOI:** 10.1371/journal.pone.0104254

**Published:** 2014-09-16

**Authors:** Célina Roda, Ioannis Nicolis, Isabelle Momas, Chantal Guihenneuc

**Affiliations:** 1 Laboratoire Santé Publique et Environnement, EA 4064, Faculté de Pharmacie, Université Paris Descartes, Sorbonne Paris Cité, Paris, France; 2 Mairie de Paris, Direction de l′Action Sociale de l′Enfance et de la Santé, Cellule Cohorte, Paris, France; University of Louisville, United States of America

## Abstract

Missing data are unavoidable in environmental epidemiologic surveys. The aim of this study was to compare methods for handling large amounts of missing values: omission of missing values, single and multiple imputations (through linear regression or partial least squares regression), and a fully Bayesian approach. These methods were applied to the PARIS birth cohort, where indoor domestic pollutant measurements were performed in a random sample of babies' dwellings. A simulation study was conducted to assess performances of different approaches with a high proportion of missing values (from 50% to 95%). Different simulation scenarios were carried out, controlling the true value of the association (odds ratio of 1.0, 1.2, and 1.4), and varying the health outcome prevalence. When a large amount of data is missing, omitting these missing data reduced statistical power and inflated standard errors, which affected the significance of the association. Single imputation underestimated the variability, and considerably increased risk of type I error. All approaches were conservative, except the Bayesian joint model. In the case of a common health outcome, the fully Bayesian approach is the most efficient approach (low root mean square error, reasonable type I error, and high statistical power). Nevertheless for a less prevalent event, the type I error is increased and the statistical power is reduced. The estimated posterior distribution of the OR is useful to refine the conclusion. Among the methods handling missing values, no approach is absolutely the best but when usual approaches (e.g. single imputation) are not sufficient, joint modelling approach of missing process and health association is more efficient when large amounts of data are missing.

## Introduction

In epidemiological studies, accurate estimate of exposure is very important for assessment of health risk. However missing data are often unavoidable, resulting from loss to follow-up in longitudinal studies, or non-responses in questionnaires. In large scale studies, certain strategies have been developed reducing the high cost of environmental measurements, e.g. collecting exposure surrogates for all subjects generally by questionnaires, and performing exact personal or environmental measurements only in subsamples of population [1]; most of the time these subsamples are small due to economic and logistic reasons (high cost, noise, bulk samplers…). For instance, in the National health and nutrition examination survey (NHANES), personal exposure to volatile organic compounds was assessed in a subsample of 851 adults, i.e. 8.5% of the population study [2]. Furthermore, even these specific studies can suffer from missing data due to measuring instrument failure, routine maintenance of monitors, and human error. Whatever the reasons for incomplete data, it can be a significant obstacle for researchers.

Most statistical software omits records with missing values by default, and analysis is conducted on a subset with the available data. This approach is commonly used for handling missing data, but can lead to loss of statistical power which can be problematic in environmental surveys where associations between environmental factors and health outcome are generally weak. Furthermore, the results of the complete cases analyses are imprecise, given that part of the data is not considered.

An alternative is to use measurements issued from subsamples to build predictive models and then, apply them to the whole population. Among these imputation techniques, single imputation approach is the most common and easily conducted as standard methodology [3], it involves a single estimated value for each missing value. It can be applied directly without loss of power due to the sample size being brought back to its original size. However, single imputation ignores the uncertainty of estimation due to the imputation.

Consequently, in 1987, Rubin proposed multiple imputation [4], where each missing value is imputed by multiple simulated data leading to multiple “completed datasets”. Each generated dataset is analyzed by standard methodology and the results combined, enabling the uncertainty attached to missing data to be assessed. Whilst several authors have declared multiple imputation their method of choice [5], [6], a recent review has suggested that its use is still quite rare: less than 2% of papers published in epidemiology journals have used multiple imputation and often omitting important details of the methodology used [7].

Another more recent alternative for dealing with missing data is to jointly model through a Bayesian approach the missing process and the association between health outcome and covariates [8], [9]. This kind of models is a part of more general approaches referred to hierarchical Bayesian modelling [10] which combines several sub-models. In our case, there are two sub-models where the first one connects missing exposure and predictive factors, and the second one assesses the association between health outcome and covariates including missing exposure. These two sub-models could be implemented separately but the estimated exposure by the first sub-model would be used in the second sub-model of disease as if it had been measured without uncertainty. Through the Bayesian modelling, the sub-models are integrated together allowing to take into account all uncertainty.

The aim of this paper is to examine performances of several approaches for handling large amounts of missing data in environmental epidemiological surveys when the data are missing completely at random (MCAR) using both a case study and a simulation study. Omitting missing values, imputation techniques (single and multiple imputation) and fully Bayesian approach are considered.

## Materials and Methods

In order to compare results from methods for handling missing values, a real dataset was used. In the PARIS (Pollution and Asthma Risk: an Infant Study) cohort, measurements were performed and pollutant levels, such as formaldehyde levels, are available in a subset of the population, but missing for infants not involved in the environmental investigation [11].

### Data

The cohort enrolled 3 840 full-term healthy babies, recruited between February 2003 and June 2006. The study protocol is described elsewhere [12]. At birth, an interview with the mother was conducted to collect data about the history of allergic conditions in both parents. Gender, parity, anthropometric parameters of the child, maternal history of pregnancy and delivery were also registered from newborn's and mother's medical records. Parents regularly documented health outcomes in mailed questionnaires. The health outcomes of interest were defined by the occurrence of lower respiratory infection (LRI) and a dry night cough (DNC) during the first twelve months of life [11], [13].

Concerning environmental and lifestyle data, a trained interviewer interviewed parents by phone during maternity leave to describe in detail home characteristics (construction date, number of occupants, home surface area, heating and cooking appliances, presence of mechanical ventilation and double glazing, wall and floor coverings and signs of dampness) and family living conditions (duration of breastfeeding, information on day-care attendance, keeping of pets, aeration, smoking, use of air fresheners, do-it-yourself (DIY)). Any changes were assessed by mailed questionnaires at each time points.

Aldehyde air sampling measurements were performed in a random sample of 196 babies'dwellings using a passive sampler [14]. Predictive factors of formaldehyde levels were previously identified: sources (presence and age of wall coating, wood-pressed products for flooring or varnished parquet floor, and particle board furniture), parameters of aeration and air stuffiness (length of window opening, presence of mechanical ventilation and double glazing), and home characteristics (construction date, housing area, and number of occupants) [11].

This study was approved by the National Ethics Committee (permissions 031153 and 051289), and parents of participating infants gave their written informed consent. Data are stored in the Paris council, within its Social, Childhood and Health Direction (DASES). All data were anonymized before statistical analysis.

### Association between formaldehyde, including missing values, and health outcomes in the PARIS cohort

In the context of the study of formaldehyde exposure impact (variable including missing values) on LRI (a relative common health outcome in infancy) or DNC (a less prevalent event), results from methods for handling missing values were compared. All infants who move during the first year of life and those with no data on health outcome were excluded from the analyses.

Unmeasured formaldehyde values are assumed MCAR [15] since families where measurements were carried out were selected at random, values are missing by design. The methods for dealing with missing data that we considered were: omitting missing data, imputation methodologies, and a fully Bayesian approach. For the first one, as its name implies, only cases with available information are considered, with cases with missing data being discarded. Concerning the imputation approach, missing data are imputed from the available information. Whatever the choice between single or multiple, an imputation model has to be established. Two approaches imputing missing formaldehyde values were examined, the linear regression model (LM), and the partial least squares (PLS) model. PLS regression is particularly suited when there are more predictors variables than observations, and contrary to LM, it allows multicolinearity between variables [16]. PLS method is based on the reduction of predictor variables dimension by using techniques near principal component analysis. As recommended in the literature, the imputation model includes variables that are used in subsequent analyses such as the outcome [17], [18]. Therefore, the imputation model included formaldehyde predictive factors and LRI or DNC. The predicted formaldehyde mean was used in the single imputation. Note that other approaches exist for single imputation where, for instance, the missing value is replaced by local or adaptative estimate [19], [20], [21] and not by the global mean. Whatever the chosen technique, the missing value is always replaced by a single value underestimating the variability due to this estimation. As the health outcomes are binary variables, the association between formaldehyde levels and LRI or DNC was then examined using logistic regression whatever the imputed model approach.

In the multiple imputation approach, several imputations are generated for given missing data. As previously described by Little and Rubin [22], “m completed datasets” were firstly created by filling in the missing values through the imputation model: missing formaldehyde values were imputed randomly from an approximate predictive distribution based on the fitted regression. For example in the case of LM, regression coefficients were sampled from their multivariate Gaussian distribution obtained on observed data and then missing formaldehyde values were replaced by their corresponding predicted values. This procedure was repeated m times. Here 10 000 imputed datasets were fitted. The completed datasets were analyzed separately, the association between formaldehyde levels (observed and imputed) and health was examined using logistic regression, and the results of all datasets were then combined, applying Rubin's rules, to yield final inference on the parameters of interest. The variance for the combined parameter estimates included between and within imputation variation.

Finally, a fully Bayesian model was implemented, as suggested by Carpenter and Kenward [18]. Two sub-models were fitted jointly using Markov Chain Monte Carlo methods [17]. The first one modelled the association between health indicator and exposure, and the second one modelled the relation between missing and observed exposure measurements including covariates supposed to be linked to exposure. Such joint modelling has advantages as mutually enhanced estimates precision of two sub-models parameters, extending multiple imputation methodology. The algorithm was run for 10 000 iterations with 1000 iterations discarded for burn-in. Inspection of posterior time series plots for the parameters as well as autocorrelation plots indicated that the model mixed well. For each model, posterior mean of OR with 95% credibility interval (95% Cr) is shown for the formaldehyde exposure. Note that since the PLS approach is not based on a probability model, Bayesian modelling cannot be used.

### Simulation study

Facing missing data, the choice of approach is crucial in terms of the conclusion, particularly when there is a high proportion of missing data (near 94% in our case), and weak associations. Comparisons between approaches have to be based on the quality of estimates, and on the ability to conclude or not a significant association. Simulation studies with characteristics near those of real data but controlling the true value of OR without omitting the case of no association (OR = 1.0) were therefore conducted. Two cases were considered: one frequent outcome similar to LRI (named “event 1”), and a second case close to DNC (“event 2”). Sample sizes are similar to those in real data sets (n = 2 551 for event 1 and n = 2 342 for event 2).

Datasets were simulated from the following steps: *ln E ∼ Ν(X φ; σ^2^ Id)* and *Y_i_ ∼ B(π_i_)* with *logit(π_i_)  =  α + β E_i_ + γ Z_i_*, *β  =  ln OR_true_* and *i  =  1, …, n*, and where formaldehyde factors are denoted by *X*, exposure variable by *E*, covariates by *Z* and health indicator by *Y*. Exposure variable (corresponding to formaldehyde in real dataset) was simulated on a logarithmic scale from a linear model with formaldehyde predictors obtained from real data [11], and coefficients *φ* were equal to those estimated in this study. Then, the health indicator was simulated from a logistic model with the resulting formaldehyde levels and covariates from real data (coefficients *γ* associated with covariates were those estimated on real data as well as the residual variance *σ* ^2^). Formaldehyde predictors and covariates are given in Table S1.

Three different *OR_true_* (and then three *β_true_* = ln *OR_true_*) between formaldehyde and event 1 or event 2 were considered: 1.0, 1.2, and 1.4. Missing values for formaldehyde were assigned at random. A case of 95% missing values was considered. To assess the robustness of our conclusions, simulations with different missing values percentages (85%, 75% and 50%) were also conducted. For each scenario, a total of 100 datasets were generated. This number of simulations is required to obtain an estimate of the regression coefficient associated with formaldehyde exposure within 10% of its true value when missing values are omitted in the real data. Indeed, the equation given by Burton et al. [23] for the number of simulations *B* is 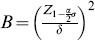 here *δ* is the specified level of accuracy of the estimate of interest “accepted”, *σ*, the standard error for the parameter of interest and, *Z_1-α/2_*, the (*1-α/2*) quantile of the standard normal distribution. When *B* = 100, *α* = 0.05 and *σ* ^2^ = 0.26 (estimated variance on real data set), *δ* is equal to 10%.

The quality of estimates for the different approaches was assessed by the root mean square error of beta coefficients (RMSE, 
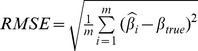
 where 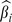 is estimated 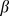 on 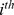 dataset 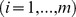 and 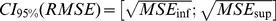 where 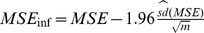 and 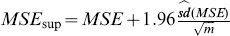 and 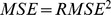). The proportion of “significant” associations (PS, 
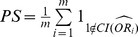
 where 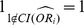, if 
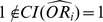
 and 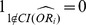, otherwise) i.e. of confidence or credibility interval of OR excluding 1 was also calculated. This criterion assesses risk of type I error when *OR_true_* = 1, and statistical power when *OR_true_* was different from 1.

Confidence intervals for RMSE were based on a Gaussian approximation using the empirical standard deviation of the MSE, and confidence intervals for the PS were based on the Clopper-Pearson “exact” confidence intervals [24] avoiding the Gaussian approximation. As the question is basically if formaldehyde exposure increased LRI and DNC risk according to previous studies in literature, the one-sided approach was chosen. Statistical analyses were conducted using R 2.14.0 [25] and WinBUGS software [26].

## Results

### Results on real data

In this study, 2 551 infants of the PARIS birth cohort were completely observed, independently of health outcomes and formaldehyde levels, and pollutant levels were available for 142 of them. Most of infants lived in apartments. Nearly 30% of buildings were built after 1975, and two-thirds were equipped with double glazing. Around half of babies had wood-pressed products for flooring or varnished parquet floor in their bedroom. Recent (less than one year old) particle board furniture was present in 49.8% dwellings. About half of infants (46.9%) had at least one LRI during their first year of life and the prevalence of DNC was 14.9%.

OR estimates between LRI or DNC occurrence and formaldehyde exposure levels are given in Table 1. The single imputation techniques (LM or PLS) clearly induced high estimates of association compared to all other approaches. The estimated OR with fully Bayesian approach was similar to that obtained with multiple imputation particularly for LRI, but lower than that with PLS imputation. However, intervals were different leading to a significant association with Bayesian modelling for LRI and nearly significant for DNC but not significant with both multiple imputation.

**Table 1:** Associations between environmental risk factor^a^ including missing values, and health outcomes, by different methods handling missing values (OR [95% CI or 95% Cr]).

		LRI^b^	DNC^c^
**Na omitted**		1.11 [0.55, +∞)	1.31 [0.45, +∞)
**Single imputation**	LM	1.91 [1.53, +∞)	5.63 [3.69, +∞)
	PLS^d^	3.27 [1.61, +∞)	3.69 [2.57, +∞)
**Multiple imputation**	LM	1.28 [0.91, +∞)	1.35 [0.14, +∞)
	PLS^d^	2.81 [0.35, +∞)	2.69 [0.39, +∞)
**Bayesian approach**		1.27 [1.10, +∞)	1.16 [0.95, +∞)

Abbreviations: CI, 95% confidence interval; Cr, 95% credibility interval, LM, linear regression model; OR, odds ratio; PLS, partial least squares.

a : Environmental factor: formaldehyde exposure, expressed in µg/m^3^, (for one unit increase in the logarithmic scale), and health outcome: lower respiratory infection (LRI) or dry nigh cough (DNC).

b : Association was adjusted for gender, socio-economic status, siblings, parental history of asthma, breastfeeding, daycare attendance, pre/postnatal tobacco smoke exposure, sign(s) of dampness, and presence of pets at home.

c : Association was adjusted for gender, socio-economic status, parental history of allergy, breastfeeding, pre/postnatal tobacco smoke exposure, gas heating, cockroaches, infant's mattress age, family events, and number of episodes of lower respiratory infections.

d : PLS imputation with two components.

### Simulations results

From the 100 simulated datasets, the resulting mean prevalence of event 1 was 29.6% (range: 18.4%–41.3%) and of event 2 was 12.3% (range: 6.8%–18.5%). Table 2 shows RMSE and PS assessed on simulated data when no data are missing. Results were quasi similar for all approaches. As RMSE depends on *OR_true_* and on prevalence of event, it increased between events 1 and 2 and slightly with *OR_true_*. Concerning event 1, frequentist and Bayesian approaches always concluded a significant association when *OR_true_* = 1.4 while when *OR_true_* = 1.2, statistic power was near 60%. For an infrequent event as event 2, statistical power decreased being near 85% when *OR_true_* = 1.4 and near 40% when *OR_true_* = 1.2. When *OR_true_* = 1.0, PS had to be equal to 5%, which was the case for the two approaches even if there was a slight increase for the less frequent event. These results can be considered as reference results as no data are missing.

**Table 2:** Root mean square error, and proportion of “significant” association with 95% confidence interval or credibility interval, on 100 replicates with no missing values.

		OR = 1.0		OR = 1.2		OR = 1.4	
		event 1	event 2	event 1	event 2	event 1	event 2
**RMSE**	**Frequentist**	0.10 [0.09, 0.11]	0.17 [0.15, 0.19]	0.10 [0.09, 0.11]	0.12 [0.11, 0.13]	0.09 [0.08, 0.10]	0.12 [0.11, 0.13]
	**Bayesian**	0.10 [0.10, 0.11]	0.17 [0.15, 0.19]	0.10 [0.08, 0.11]	0.12 [0.10, 0.14]	0.09 [0.08, 0.11]	0.12 [0.11, 0.14]
**PS**	**Frequentist**	5 [1.6, 11.3]	8 [3.5, 15.2]	59 [48.7, 68.7]	40 [30.3, 50.2]	100	83 [74.2, 89.8]
	**Bayesian**	4 [1.1, 9.9]	7 [2.9, 13.9]	61 [50.7, 70.6]	39 [29.4, 49.3]	100	86 [77.6, 92.1]

Abbreviations: RMSE, root mean square error; OR, odds ratio; PS, proportion of “significant” association.

Sample size for each simulated dataset: event 1 N = 2 551/event 2 N = 2 342.

Table 3 provides results of the RMSE on replicates with 95% of missing values. RMSE values ranged from 0.18 to 0.83 for event 1, and from 0.09 to 1.06 for event 2. For each event, RMSE slightly increased with OR. RMSE values were always lower in multiple imputation than in single imputation, whatever the imputation model (LM or PLS). Single imputation led to huge RMSE reflecting poor qualities of estimates. All results were confirmed with proportion of missing values of 75% and 85% (Table S2).

**Table 3:** Root mean square error of beta coefficients with 95% confidence interval based on 100 replicates with 95% of missing values.

		OR = 1.0		OR = 1.2		OR = 1.4	
		event 1	event 2	event 1	event 2	event 1	event 2
**Na omitted**		0.33 [0.29, 0.36]	0.11 [0.10, 0.12]	0.22 [0.20, 0.24]	0.09 [0.08, 0.10]	0.18 [0.17, 0.20]	0.17 [0.15, 0.18]
**Single imputation**	LM	0.46 [0.42, 0.50]	1.06 [0.95, 1.16]	0.57 [0.52, 0.61]	0.96 [0.89, 1.03]	0.70 [0.66, 0.74]	1.02 [0.95, 1.09]
	PLS	0.58 [0.47, 0.67]	0.89 [0.75, 1.01]	0.63 [0.52, 0.73]	0.81 [0.70, 0.91]	0.83 [0.71, 0.94]	0.99 [0.82, 1.13]
**Multiple imputation**	LM	0.30 [0.27, 0.32]	0.33 [0.30, 0.36]	0.29 [0.26, 0.32]	0.29 [0.26, 0.32]	0.30 [0.27, 0.33]	0.29 [0.26, 0.31]
	PLS	0.48 [0.40, 0.56]	0.75 [0.62, 0.85]	0.49 [0.41, 0.56]	0.66 [0.57, 0.74]	0.65 [0.57, 0.73]	0.76 [0.65, 0.86]
**Bayesian approach**		0.18 [0.16, 0.20]	0.26 [0.23, 0.29]	0.18 [0.16, 0.20]	0.24 [0.20, 0.27]	0.19 [0.17, 0.22]	0.24 [0.21, 0.26]

Abbreviations: LM, linear model; OR, odds ratio; PLS, partial least squares.

Sample size for each simulated dataset: event 1 N = 2 551/event 2 N = 2 342.

Figure S1 shows boxplots of beta estimates obtained from Bayesian approach, from 100 simulations for the three different values of *OR_true_*. When *OR_true_* is equal to 1.0, the range of estimates increased with the proportion of missing values and with the decrease of event prevalence. These results were also observed when *OR_true_* = 1.2 and *OR_true_* = 1.4. Moreover, an underestimation of beta (and so of OR) was obtained when *OR_true_* = 1.4.

When *OR_true_* is equal to 1, real risk of type I error is assessed by PS while the theoretical one was fixed at 5% (Table S3). For event 1 with 95% of missing data, single imputation led to very high risks (23% [15.2, 32.5] and 21% [13.5, 30.3] for LM and PLS, respectively). If missing proportion is 75% or 85%, huge risks of type I error were again found for single imputation. For event 2, PS considerably increased for single imputation, 35% [25.7, 45.2] and 42% [32.2, 52.3] for LM and PLS, respectively. This increase was confirmed with 75% and 85% of missing values. Multiple imputation led to always conservative results explained by large confidence intervals of OR obtained with this approach whether for event 1 or 2. Bayesian approach led to increase risk of type I error. For event 1, this increase seems reasonable because 5% is always in the confidence interval (7% [2.9, 13.9] with 95% missing values). For the infrequent event 2, risk of type I error increases and excluding 5% from confidence intervals when 85% and 95% of data are missing (e.g. 16% [9.4, 24.7] with 95% missing values).

**Figure 1:**
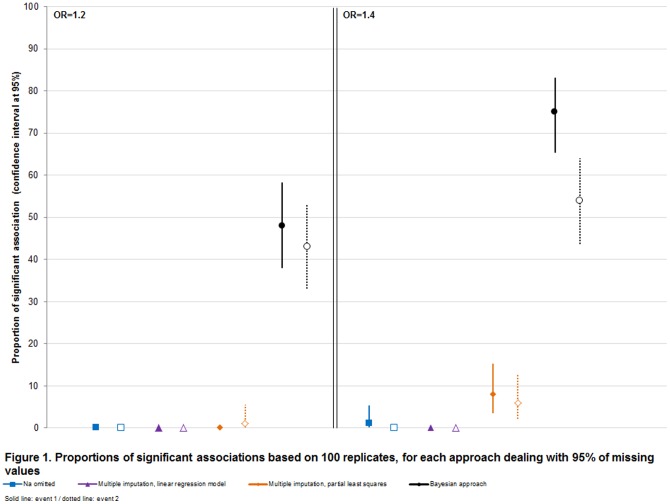
Proportions of significant associations based on 100 replicates, for each approach dealing with 95% missing values.

Figure 1 presents PS when *OR_true_* is greater than 1 and 95% of missing values for two events and for all approaches, excluding single imputation which had given a weak quality on estimates and overestimated risk of type I error. As expected, statistical power increased with *OR_true_* and decreased for infrequent event. Weak performances were obtained for multiple linear and PLS imputation. This figure clearly shows highest PS for Bayesian model near reference power on complete data (Table 1) especially for event 1, all other approaches giving a null or quasi null power for *OR_true_* = 1.2 and *OR_true_* = 1.4, respectively. Figure S2 presents PS when *OR_true_* is greater than 1 for 85% and 75% of missing values. Highest PS were clearly obtained for Bayesian model.

And with 50% of missing data, statistical power increased remaining the best for Bayesian approach especially for infrequent event (near 80% by Bayesian approach against 53% by LM multiple imputation when *OR_true_* = 1.4).

## Discussion

This paper addresses the crucial question of how to handle large amounts of missing data in environmental epidemiological surveys. Till now, as far as we know, very few teams have compared performances of approaches handling missing values with a proportion of missingness above 75% [27], [28]. To solve this question, we used both real and simulated data to determine the most appropriate approach when there is a large amount of missing data. Results on RMSE and PS showed poor performances with single imputation. Fully Bayesian approach seems better, followed by imputation approaches, which in turn gave better results than omitting missing observations.

As expected, we found that omitting missing values is less efficient than single and multiple imputations. Even if it is the easiest approach for handling missing values, it should be used only in presence of less than 5% of missing values [29] because it should induce a significant loss of statistical power: unrealistic when the health outcome is infrequent and problematic in environmental studies where pollutants have often a weak impact.

Another common approach for handling missing values is to impute them before any analysis which is commonly used in environmental epidemiological studies to estimate exposure levels for all study members. An increase in power is the substantial benefit of this alternative over omitting missing values. Nevertheless, the specification of the imputation model is an important step. As previously demonstrated, if the imputation model is not properly specified the imputation approach could introduce a bias which is not present in omitting missing values when missingness is MCAR [28]. It has been observed that including many relevant variables in the imputation model tends to make the missing at random assumption more plausible [30], even if computational problems could occur such as multicollinearity and a large number of predictors might provide instable estimates. Previous authors showed the importance of including the health outcome because regression coefficients came close to the truth [31], [32]. Note that inclusion of the health outcome implies that the imputation procedure has to be renewed for each new health outcome.

Single imputation is easy to implement, but the major disadvantage is the overestimation of association between exposure and health outcome increasing with the strength of the association. As explained by Rubin [4], this overstatement is certainly due to that one imputed value cannot itself represent uncertainty about imputed value.

Conversely, multiple imputation takes into account the uncertainty and thus, does not underestimate the variance of estimates. But this approach is conservative. In fact, as previously described in the literature [31], large imprecision of OR estimates was observed, thus yielding to no significant associations. Moreover, concerning the choice of the number of imputations, it has been suggested [4], that less than 10 imputed datasets are useful compared with an infinite number of imputations. However, when the percentage of missing values is huge, more than 10 imputations may be needed and the number of imputed data should approximate the number of observations with missing data, as previously suggested [33]. Although 2 000 imputations could have been sufficient in this study, we fitted 10 000 imputed datasets as a precaution even if it was time consuming. Bayesian joint modelling appears to be less conservative with a statistical power near 75% for event 1 (near 55% for the infrequent event 2) when *OR_true_* = 1.4 with 95% of missing data. Nevertheless, it is important to notice that risk of type I error tends to increase, slightly for event 1 (near 7%) and much more for the infrequent event 2 (near 16%). Even if the RMSE is always smaller for the Bayesian approach compared to the other approaches and indicates a better performance in terms of estimates, boxplots of the beta estimates from 100 simulations under *OR_true_* = 1.0 clearly show that the range of estimates increases with decrease of event prevalence and with increase in the missing values proportion. This result is confirmed when OR is greater than one but a bias appears when *OR_true_* increases. Thus, caution should be taken when interpreting results for an infrequent event with high proportion of missing values. In addition, the use of empirical approaches (e.g. bootstrap, Monte Carlo study) could be useful to assess the real risk of type I error.

For infrequent events (for instance with prevalence less than 10%) and high proportion of missing values, statistical power remains too weak. Bayesian approach offers the possibility to obtain easily estimated posterior distribution of OR which could be a useful tool to refine conclusions. Posterior probability of OR being smaller than 1 can indeed be deduced and such probability between 5% and 10% could be considered weak enough to conclude an “almost significant” association. This strategy would lead to three possible conclusions: “not significant”, “significant” or “almost significant”. Thus, for event 2, the classical approach yields 46 non significant associations when *OR_true_* = 1.4, but among them, 7 would be declared as “almost significant”. It is noteworthy that the proportion of non significant associations now labeled “almost significant” increases with *OR_true_* from 8.8% (*OR_true_* = 1.2) to 15.2% (*OR_true_* = 1.4). If the type I error is assessed only among associations not declared “almost significant”, it remains stable (16% and 17.8% for classical and this new strategy, respectively).

In conclusion, among the methods dealing with missing data, no approach is absolutely better than the others in all circumstances. In the presence of high proportions of missing values, using only complete data yields to a significant loss of statistical power. Single imputation underestimates the variance, thus overestimating the association between environmental factor and health outcome. Multiple imputation, due to overcoverage, is too conservative and unable to show significant associations. When the health outcome is frequent, joint modelling seems to be more efficient than other approaches, combining low RMSE, limited increase of risk of type I error, and high statistical power. The simulation study is useful for explaining the disparity of associations found in the real data, for example for LRI (Table 1) corresponding to a frequent event. Indeed, the characteristics of each method highlighted by the simulation study are found in the real case, i.e. bias using simple imputation, lack of power using multiple imputation, and significant association using Bayesian approach. The conclusion of a significant association between formaldehyde exposure and LRI is strengthened. With regards to the infrequent event, DNC, only a tendency of an association is observed. No approach gives completely satisfactory results when the health outcome is infrequent and the proportion of missing values is high. Though the Bayesian modelling has the best power and precision of estimates, this comes at a cost of inflated risk of type I error. However, estimated posterior distribution of OR would be helpful to refine the conclusion by introducing a new category of “almost significant association” when probability of OR less than 1 is between 5% and 10%. Concerning inflation of risk of type I error, correcting methodology as bootstrap could be implemented. This would lead certainly to very huge computer time as MCMC iterative algorithm would be used on each bootstrapped sample. An alternative approach to MCMC in repeated Bayesian estimations such as “Importance Sampling” could be envisaged [34].

## Supporting Information

Figure S1 **Boxplots of** ***β*** **(** ***β  =  ln OR*** **) estimates under Bayesian approach from 100 simulated datasets for the three different values of true OR dealing with no missing values, 75%, 85% and 95% of missing values.**
(PDF)

Figure S2 **Proportions of significant associations based on 100 replicates, for each approach dealing with 85%, and 75% of missing values.**
(PDF)

Table S1 **Predictive factors of formaldehyde exposure and covariates used for adjustment in the model relating formaldehyde exposure and health indicator.**
(DOC)

Table S2 **Root mean square error of beta coefficients with 95% confidence interval based on 100 replicates with 85%, and 75% of missing values.**
(DOC)

Table S3 **Proportions of significant associations with 95% confidence interval based on 100 replicates with 95%, 85% and 75% of missing values when** ***OR_true_*** **= 1.**
(DOC)
