# Supplementary material for: New Insights into Handling Missing Values in Environmental Epidemiological Studies
Source: PLoS One. 2014 Sep 16;9(9):e104254. doi: 10.1371/journal.pone.0104254 (PMC4165576; doi:10.1371/journal.pone.0104254)
Supplement: Figure S1 — Boxplots of β ( β = ln OR ) estimates under Bayesian approach from 100 simulated datasets for the three different values of true OR dealing with no missing values, 75%, 85% and 95% of missing values. (PDF) [file pone.0104254.s001.pdf]

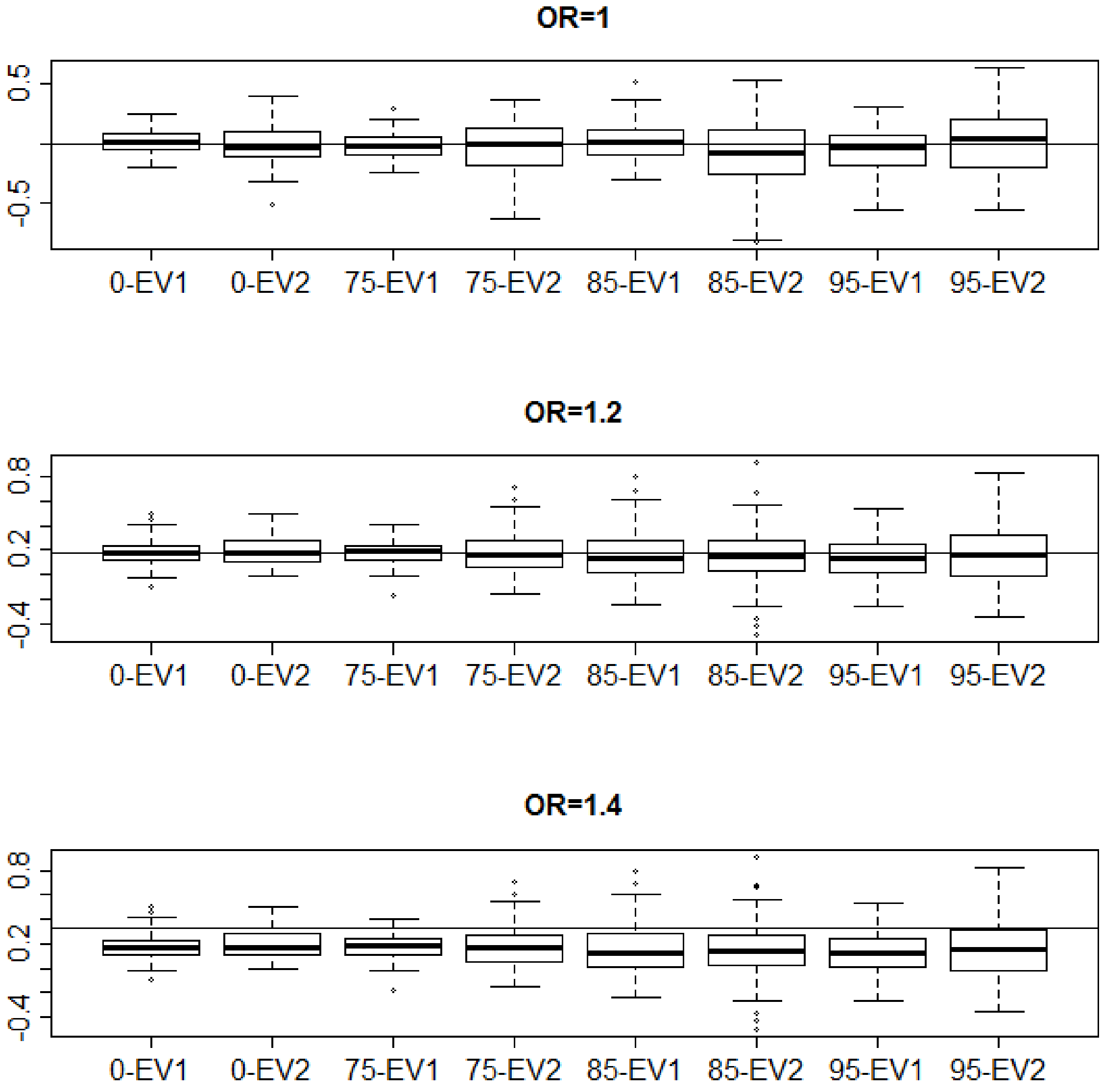

Figure S1. Boxplots of β (β = ln(OR)) estimates under Bayesian approach from 100 simulated datasets for the three different values of true OR dealing with no missing values, 75%, 85% and 95% of missing values.

Abbreviations: x-EVy, x% of missing values for event y
