# Supplementary material for: New Insights into Handling Missing Values in Environmental Epidemiological Studies
Source: PLoS One. 2014 Sep 16;9(9):e104254. doi: 10.1371/journal.pone.0104254 (PMC4165576; doi:10.1371/journal.pone.0104254)
Supplement: Figure S2 — Proportions of significant associations based on 100 replicates, for each approach dealing with 85%, and 75% of missing values. (PDF) [file pone.0104254.s002.pdf]

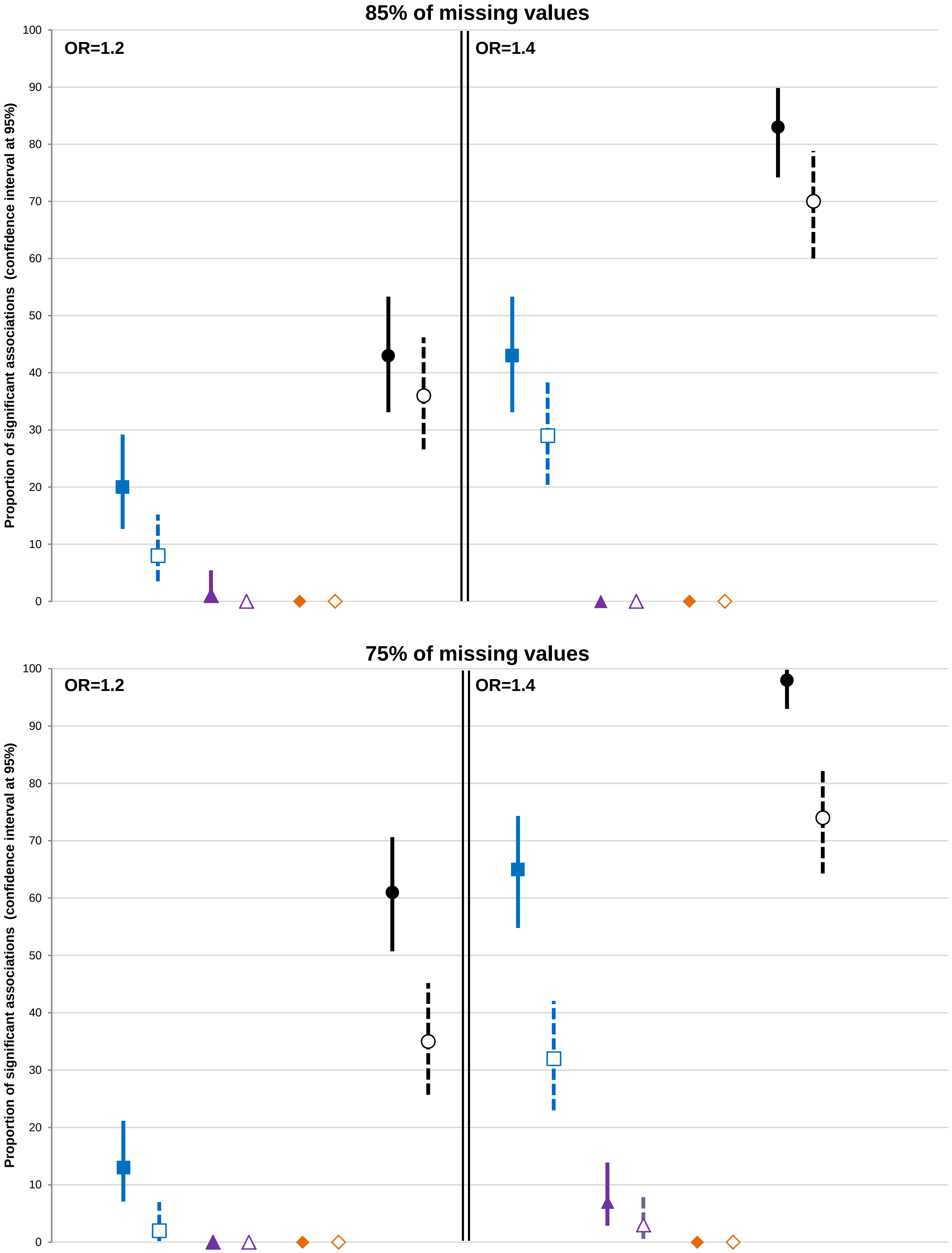

Figure S2. Proportion of significant associations based on 100 replicates, for each approach dealing with 85%, and 75% of missing values

Na omitted   Multiple imputation, linear regression model   Multiple imputation, partial least squares   Bayesian approach

Solid line: event 1 / dotted line: event 2
