# Supplementary material for: New Insights into Handling Missing Values in Environmental Epidemiological Studies
Source: PLoS One. 2014 Sep 16;9(9):e104254. doi: 10.1371/journal.pone.0104254 (PMC4165576; doi:10.1371/journal.pone.0104254)
Supplement: Table S1 — Predictive factors of formaldehyde exposure and covariates used for adjustment in the model relating formaldehyde exposure and health indicator. (DOC) [file pone.0104254.s003.doc]

**Table S1.** Predictive factors of formaldehyde exposure and covariates used for adjustment in the model relating formaldehyde exposure and health indicator.

| **Predictive factors** | **Covariates** | **LRI** | **DNC** |
| --- | --- | --- | --- |
| Construction date: after 1990, 1976-1990, before 1975 | Gender: male, female | X | X |
| Housing area: ≥ 70m², < 70m² | Socio-economic status: low, medium, high level | X | X |
| Number of occupants: > 3, ≤ 3 | Parental history of asthma: yes, no | X |  |
| Wall coating (paint or fiber cloth): yes ≥ 1 year, yes < 1 year, no | Parental history of allergy: yes, no |  | X |
| Wood-pressed products for flooring or varnished parquet floor: yes ≥ 1 year, yes < 1 year, no | Breastfeeding: ≥ 3 months, < 3 months | X | X |
| Particle board furniture: yes ≥ 1 year, yes < 1 year, no | Siblings: ≥ 2, < 2 | X |  |
| Mechanical ventilation: yes, no | Day-care attendance: yes, no | X |  |
| Double gazing: yes, no | Furry pets: yes, no | X |  |
| Duration of window opened: 🠙 1 hour | Sign(s) of dampness: ≥ 2 signs, 1 sign, no | X |  |
| Season of declaration (duration of window opened): cold season, hot season | Infant’s mattress age: used, new |  | X |
|  | Cockroaches: yes, no |  | X |
|  | Family stressor events: yes, no |  | X |
|  | Gas heating: yes, no |  | X |
|  | Prenatal exposure to ETS: yes, no | X | X |
|  | Postnatal exposure to ETS: yes, no | X | X |
|  | LRI: number of episodes |  | X |

Abbreviations: DNC: dry night cough, ETS, environmental tobacco smoke, LRI: lower respiratory infections
