# Supplementary material for: New Insights into Handling Missing Values in Environmental Epidemiological Studies
Source: PLoS One. 2014 Sep 16;9(9):e104254. doi: 10.1371/journal.pone.0104254 (PMC4165576; doi:10.1371/journal.pone.0104254)
Supplement: Table S2 — Root mean square error of beta coefficients with 95% confidence interval based on 100 replicates with 85%, and 75% of missing values. (DOC) [file pone.0104254.s004.doc]

**Table S2.** Root mean square error of beta coefficients with 95% confidence interval based on 100 replicates with 85%, and 75% of missing values.

|  | | **85% of missing values** | | | | | | |
| --- | --- | --- | --- | --- | --- | --- | --- | --- |
|  |  | **OR=1.0** | | **OR=1.2** | | **OR=1.4** | | |
|  |  | **event 1** | **event 2** | **event 1** | **event 2** | | **event 1** | **event 2** |
| **Na omitted** |  | 0.23 [0.20, 0.25] | 0.48 [0.44, 0.53] | 0.25 [0.22, 0.27] | 0.34 [0.31, 0.37] | | 0.23 [0.21, 0.25] | 0.35 [0.32, 0.38] |
| **Single imputation** | LM | 0.42 [0.37, 0.46] | 1.27 [1.14, 1.39] | 0.60 [0.55, 0.65] | 1.10 [1.01, 1.18] | | 0.71 [0.68, 0.74] | 1.27 [1.19, 1.34] |
|  | PLS | 0.44 [0.37, 0.51] | 0.91 [0.78, 1.01] | 0.62 [0.51, 0.72] | 0.80 [0.66, 0.93] | | 0.74 [0.66, 0.81] | 0.89 [0.77, 1.00] |
| **Multiple imputation** | LM | 0.28 [0.25, 0.31] | 0.52 [0.47, 0.57] | 0.40 [0.36, 0.43] | 0.35 [0.31, 0.38] | | 0.40 [0.36, 0.42] | 0.47 [0.43, 0.50] |
|  | PLS | 0.42 [0.35, 0.48] | 0.85 [0.74, 0.95] | 0.59 [0.48, 0.68] | 0.75 [0.62, 0.87] | | 0.69 [0.62, 0.75] | 0.83 [0.71, 0.93] |
| **Bayesian approach** |  | 0.14 [0.11, 0.16] | 0.32 [0.27, 0.36] | 0.20 [0.17, 0.23] | 0.24 [0.19, 0.27] | | 0.18 [0.15, 0.20] | 0.23 [0.20, 0.26] |
|  |  |  |  |  |  | |  |  |
|  | | **75% of missing values** | | | | | | |
|  |  | **OR=1.0** | | **OR=1.2** | | | **OR=1.4** | |
|  |  | **event 1** | **event 2** | **event 1** | **event 2** | | **event 1** | **event 2** |
| **Na omitted** |  | 0.13 [0.11, 0.14] | 0.23 [0.21, 0.25] | 0.11 [0.09, 0.12] | 0.16 [0.14, 0.18] | | 0.11 [0.10, 0.12] | 0.17 [0.15, 0.19] |
| **Single imputation** | LM | 0.31 [0.27, 0.34] | 0.77 [0.69, 0.84] | 0.44 [0.42, 0.46] | 0.78 [0.73, 0.83] | | 0.68 [0.66, 0.69] | 0.96 [0.93, 1.00] |
|  | PLS | 0.27 [0.24, 0.31] | 0.48 [0.41, 0.53] | 0.36 [0.32, 0.40] | 0.45 [0.37, 0.51] | | 0.54 [0.48, 0.58] | 0.51 [0.45, 0.57] |
| **Multiple imputation** | LM | 0.19 [0.17, 0.21] | 0.35 [0.31, 0.84] | 0.19 [0.17, 0.21] | 0.31 [0.28, 0.33] | | 0.25 [0.24, 0.26] | 0.31 [0.29, 0.33] |
|  | PLS | 0.27 [0.23, 0.30] | 0.46 [0.40, 0.52] | 0.35 [0.31, 0.39] | 0.43 [0.35, 0.49] | | 0.51 [0.46, 0.56] | 0.49 [0.43, 0.55] |
| **Bayesian approach** |  | 0.11 [0.09, 0.12] | 0.21 [0.18, 0.24] | 0.10 [0.08, 0.12] | 0.16 [0.14, 0.19] | | 0.10 [0.08, 0.12] | 0.17 [0.14, 0.20] |
|  |  |  |  |  |  | |  |  |

Abbreviations: LM, linear model; OR, odds ratio; PLS, partial least squares

Sample size for each simulated dataset: event 1 N=2 551 / event 2 N=2 42
