# Supplementary material for: New Insights into Handling Missing Values in Environmental Epidemiological Studies
Source: PLoS One. 2014 Sep 16;9(9):e104254. doi: 10.1371/journal.pone.0104254 (PMC4165576; doi:10.1371/journal.pone.0104254)
Supplement: Table S3 — Proportions of significant associations with 95% confidence interval based on 100 replicates with 95%, 85% and 75% of missing values when ORtrue = 1. (DOC) [file pone.0104254.s005.doc]

**Table S3.** Proportions of significant associations with 95% confidence interval based on 100 replicates with 95%, 85% and 75% of missing values when *OR_true_* = 1.

| **Proportion of missing values** | | **95%** | | **85%** | | **75%** | |
| --- | --- | --- | --- | --- | --- | --- | --- |
|  |  | **event 1** | **event 2** | **event 1** | **event 2** | **event 1** | **event 2** |
| **Na omitted** |  | 0 | 0 | 1 [0.03, 5.4] | 2 [0.2, 7.0] | 0 | 0 |
| **Single imputation** | LM | 23 [15.2, 32.5] | 35 [25.7, 45.2] | 27 [18.6, 36.8] | 30 [21.2, 40.0] | 17 [10.2, 25.8] | 25 [16.9, 34.7] |
|  | PLS | 21 [13.5, 30.3] | 42 [32.2, 52.3] | 33 [23.9, 43.1] | 24 [16.0, 33.6] | 15 [8.6, 23.5] | 21 [13.5, 30.3] |
| **Multiple imputation** | LM | 0 | 0 | 0 | 0 | 0 | 0 |
|  | PLS | 0 | 0 | 0 | 0 | 0 | 0 |
| **Bayesian approach** |  | 7 [2.9, 13.9] | 16 [9.4, 24.7] | 9 [4.2, 16.4] | 13 [7.1, 21.2] | 8 [3.5, 15.2] | 7 [2.9, 13.9] |

Abbreviations: LM, linear model; OR, odds ratio; PLS, partial least squares

Sample size for each simulated dataset: event 1 N=2 551 / event 2 N=2 342
